# Treating Acute Severe Eosinophilic Asthma with IL-5 Inhibitors in ICU

**DOI:** 10.1155/2022/2180795

**Published:** 2022-08-21

**Authors:** Nicolas Barbarot, Emmanuelle Nourry, Nicolas Massart, François Legay, Matthieu Debarre, Pierre Fillatre, Eric Magalhaes, Arnaud Mari, Julien Wallois, Eric Briens, Stéphane Jouneau

**Affiliations:** ^1^Service de Réanimation, CH de SAINT BRIEUC, 10 rue Marcel Proust 22000 Saint-Brieuc, France; ^2^Service de Pneumologie, CH de SAINT BRIEUC, 10 rue Marcel Proust 22000 Saint-Brieuc, France; ^3^Service de Pneumologie, CHU de Rennes, 2 rue Henri Le Guilloux 35033 Rennes, France; ^4^IRSET UMR 1085, Université de Rennes 1, 35043 Rennes, France

## Abstract

**Introduction:**

About 10% of the 300 million people worldwide who suffer from asthma have a severe disease that is uncontrolled despite treatment with inhaled corticosteroids and long-acting beta agonists. The eosinophilic inflammation pathway in the respiratory tract and blood is involved and interleukin-5 (IL-5) has recently been identified as a major promotor of this pathway. The anti-IL-5 antibodies reduce the incidence of exacerbation and allowed steroid sparing in severe asthma patients but only two case reports have been published on their use in critical care. *Case Presentation*. This report describes the extraordinary clinical improvement of a young patient with steroid-refractory eosinophilic acute severe asthma who required mechanical ventilation, VV-ECMO followed by treatment with mepolizumab. The salient point in this case is the use of an anti-IL-5 monoclonal antibody for a critically ill patient whose condition was deteriorating despite mechanical ventilation and VV-ECMO. The usual steroid treatment failed to control the increase in blood eosinophils or his bronchial inflammation and constriction.

**Conclusion:**

Anti-IL-5 antibodies are now a standard treatment for severe eosinophilic asthma that can also be useful in an emergency to treat steroid-refractory eosinophilic acute severe asthma.

## 1. Introduction

About 10% of the 300 million people worldwide who suffer from asthma have a severe disease [1] that is uncontrolled despite treatment with inhaled corticosteroids and long-acting beta agonists [2]. The eosinophilic inflammation pathway in the respiratory tract and blood is involved and interleukin-5 (IL-5) has recently been identified as a major promotor of this pathway [1]. While acute severe asthma (ASA) is life threating and requires prompt intervention [3], few patients require mechanical ventilation. Associated hospital mortality decreased over time, thanks to last resort therapy in the more severe cases such as venovenous extra corporal membrane oxygenation (VV-ECMO) [4]. The anti-IL-5 antibodies reduce the incidence of exacerbation and allowed steroid sparing in severe asthma patients [5, 6] but only two case reports have been published on their use in critical care [4, 7]. This report describes the extraordinary clinical improvement of a young patient with steroid-refractory eosinophilic ASA who required VV-ECMO followed by treatment with mepolizumab.

## 2. Case Presentation

This 31-year-old man who had not been exposed to any allergen or infectious person was first treated in the emergency department for acute-onset dyspnea. His only existing conditions were atopic dermatitis and asthma treated with inhaled salbutamol on demand without hospitalization. There was no history of sinusitis or nasal polyps. He smoked 20 to 40 cigarettes a day. At admission, he suffered from acute respiratory failure with bronchospasm, had no fever, and showed no sign of biological inflammation or hypereosinophilia (210/*μ*L). He was given 6 L/min of oxygen and arterial blood gases analysis was as follows: pH: 7.38, PaCO_2_: 45 mmHg, and PaO_2_: 75 mmHg. A chest X-ray confirmed thoracic distention. He was given methylprednisolone (2 mg/kg) and many salbutamol nebulizations and transferred to the intensive care unit (ICU) for critical ASA.

Orotracheal intubation was needed soon after admission because of respiratory distress and increased hypoxia and hypercapnia. His clinical condition worsened despite deep sedation, a neuromuscular blocking agent, aerosol therapy, intravenous steroids (1 mg/kg, 2/day), and appropriate ventilation (tidal volume 400 mL–6 mL/kg, respiration 19/min, and zero end-expiratory pressure). His respiratory acidosis became uncontrollable on day 3 (pH: 7.23, PaCO_2_: 86 mmHg, intrinsic PEEP: 12 cm H_2_0) and a femoro-jugular VV-ECMO was established.

We ruled out infection on bronchoalveolar lavage (BAL) with negative film array assessing bacteria, viruses and fungi. There were no signs of bronchopulmonary allergic aspergillosis or hypereosinophilic syndrome. Tests for antineutrophil cytoplasm antibodies were negative and bronchoscopy detected no macroscopic abnormalities. A chest CT-scan showed micronodules in the middle lobe. Lastly, BAL cytology revealed an eosinophilic predominance (64% eosinophils), and his blood eosinophil count increased to 840/*μ*L on day 7 despite treatment with methylprednisolone (1 mg/kg, 2/day).

**Figure 1:**
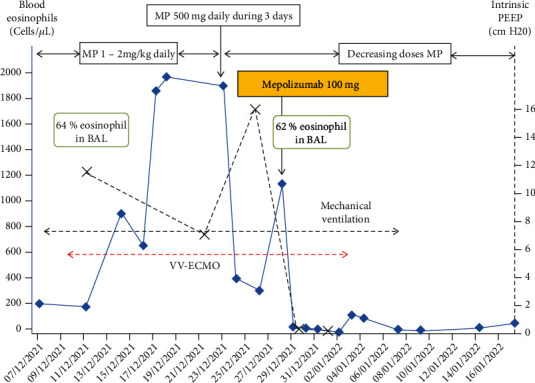
Evolution of respiratory support, intrinsic PEEP, blood, and alveolar eosinophils count over time. MP: methylprednisolone.

The steroids were then increased to 500 mg methylprednisolone per day from day 16 to day 19 without any clear clinical improvement. His bronchospasm persisted, as did his hypereosinophilia (BAL eosinophilic cells: 62% on day 20). The patient's clinical condition became serious, with VV-ECMO-related hemorrhagic complications and thrombocytopenia. He was given mepolizumab (100 mg) on day 20 and his clinical condition improved dramatically 48 hours later: the bronchospasm stopped, his intrinsic PEEP became normal and his blood eosinophil count decreased (Figure 1). The VV-ECMO was removed one week later, and the intubation removed 30 days after admission.

Pulmonary function tests (PFTs) confirmed the obstructive pattern (FEV1/FVC ratio = 0.7) that was reversed with *β*2-stimulant bronchodilator. The patient was sent home 7 days after leaving the ICU with inhaled therapy plus fluticasone/salmeterol and oral prednisone decreasing to 4 mg daily. He was encouraged to stop smoking and remained on mepolizumab (100 mg every 4 weeks). His asthma remained well controlled four months later, his blood eosinophilia was normal (70/*μ*L), and his FEV1/FVC ratio was 0.81.

## 3. Discussion

Few (about 2.4%) mechanically ventilated ASA patients die in hospital [8], perhaps because patients with isolated respiratory failure are generally young and have no comorbidities. However, salvage options should be considered for some patients. VV-ECMO was started early for this patient because of his refractory respiratory acidosis despite appropriate mechanical ventilation as recommended by the French recommendations [3]. As VV-ECMO results in many fewer deaths (6%) among these patients than for others (30-55%) [9], we believe that VV-ECMO saved our patient's life by giving us time to find an effective treatment.

The salient point in this case is the use of an anti-IL-5 monoclonal antibody for a critically ill patient whose condition was deteriorating despite mechanical ventilation and VV-ECMO. The usual steroid treatment failed to control the increase in blood eosinophils or his bronchial inflammation and constriction.

We have found only 2 descriptions of similar cases. One briefly discusses a patient with ASA and eosinophilic alveolitis on VV-ECMO who was successfully treated with an anti-IL-5 antibody [4]. The other describes an eosinophilic asthmatic patient who was mechanically ventilated for ASA whose bronchospasm and respiratory acidosis persisted despite being given prednisolone 200 mg/day for a whole week. Mepolizumab treatment produced a clinical improvement within 2 days, together with a decrease in blood eosinophils. The outcome was favorable and the patient was discharged alive [7].

Mepolizumab binds to circulating IL-5, so inhibiting the maturation of bone marrow eosinophils, decreasing their migration to the blood, and their degranulation in the respiratory tract mucosa, which decreases bronchial reactivity and secretion [10]. As it is effective for treating eosinophilic severe asthma and limits exacerbation [5], it is indicated as a maintenance therapy [1]. And mepolizumab is suitable for treating all the subtypes of eosinophilic asthma [11].

We believe that mepolizumab was a crucial treatment that rapidly normalized our patient's eosinophilia. The respiratory improvement was so evident that the VV-ECMO was removed within 5 days, followed by extubation a few days later.

The rapid action of mepolizumab is similar to that reported in the only similar published case [7], supporting its use in a critical care setting. The decrease in the eosinophil count within 2 days and the concomitant clinical improvement is consistent with published findings [12]. However, the plasma concentration of mepolizumab can increase for up to 4-8 days after subcutaneous injection. In validated indication, its clinical benefit is usually seen weeks after treatment initiation [5, 6].

In agreement with others [6], we found that the steroids and mepolizumab were complementary, as indicated by the temporary, reversible effect of the steroid bolus on the blood eosinophil count. The anti-IL-5 antibody has no effect on the mature eosinophils or their precursors in respiratory secretions. This pulmonary eosinophil maturation enables local inflammation to escape from IL-5 inhibitors [13].

In conclusion, anti-IL-5 antibodies are now a standard treatment for severe eosinophilic asthma that can also be useful in an emergency to treat steroid-refractory eosinophilic ASA.

## Data Availability

The data are available from the corresponding author upon reasonable demand.
